# Stochastic Modeling of B Lymphocyte Terminal Differentiation and Its Suppression by Dioxin

**DOI:** 10.1186/1752-0509-4-40

**Published:** 2010-04-01

**Authors:** Qiang Zhang, Sudin Bhattacharya, Douglas E Kline, Robert B Crawford, Rory B Conolly, Russell S Thomas, Norbert E Kaminski, Melvin E Andersen

**Affiliations:** 1 Division of Computational Biology, The Hamner Institutes for Health Sciences, Research Triangle Park, NC 27709, USA; 2 Department of Pharmacology & Toxicology and Center for Integrative Toxicology, Michigan State University, East Lansing, MI 48824, USA; 3 Integrated systems Toxicology Division, National Health and Environmental Effects Research Laboratory, Office of Research and Development, U.S. Environmental Protection Agency, Research Triangle Park, NC 27711, USA

## Abstract

**Background:**

Upon antigen encounter, naïve B lymphocytes differentiate into antibody-secreting plasma cells. This humoral immune response is suppressed by the environmental contaminant 2,3,7,8-tetrachlorodibenzo-p-dioxin (TCDD) and other dioxin-like compounds, which belong to the family of aryl hydrocarbon receptor (AhR) agonists.

**Results:**

To achieve a better understanding of the immunotoxicity of AhR agonists and their associated health risks, we have used computer simulations to study the behavior of the gene regulatory network underlying B cell terminal differentiation. The core of this network consists of two coupled double-negative feedback loops involving transcriptional repressors Bcl-6, Blimp-1, and Pax5. Bifurcation analysis indicates that the feedback network can constitute a bistable system with two mutually exclusive transcriptional profiles corresponding to naïve B cells and plasma cells. Although individual B cells switch to the plasma cell state in an all-or-none fashion when stimulated by the polyclonal activator lipopolysaccharide (LPS), stochastic fluctuations in gene expression make the switching event probabilistic, leading to heterogeneous differentiation response among individual B cells. Moreover, stochastic gene expression renders the dose-response behavior of a population of B cells substantially graded, a result that is consistent with experimental observations. The steepness of the dose response curve for the number of plasma cells formed vs. LPS dose, as evaluated by the apparent Hill coefficient, is found to be inversely correlated to the noise level in Blimp-1 gene expression. Simulations illustrate how, through AhR-mediated repression of the AP-1 protein, TCDD reduces the probability of LPS-stimulated B cell differentiation. Interestingly, stochastic simulations predict that TCDD may destabilize the plasma cell state, possibly leading to a reversal to the B cell phenotype.

**Conclusion:**

Our results suggest that stochasticity in gene expression, which renders a graded response at the cell population level, may have been exploited by the immune system to launch humoral immune response of a magnitude appropriately tuned to the antigen dose. In addition to suppressing the initiation of the humoral immune response, dioxin-like compounds may also disrupt the maintenance of the acquired immunity.

## Background

In response to antigen stimulation, naïve B cells residing in lymphoid organs such as the spleen and lymph nodes differentiate terminally into antibody-secreting plasma cells [1,2]. This adaptive humoral immune response can be adversely affected by exposure to some environmental chemicals [3-5]. The environmental contaminant 2,3,7,8-tetrachlorodibenzo-p-dioxin (TCDD) and other dioxin-like compounds with similar structures suppress humoral immunity, mainly by interfering with B cell differentiation and subsequent antibody secretion [5-8]. These compounds, capable of producing a variety of additional toxic responses including cancer, liver damage, and developmental defects, pose a serious potential risk to human health [9].

**Figure 1:**
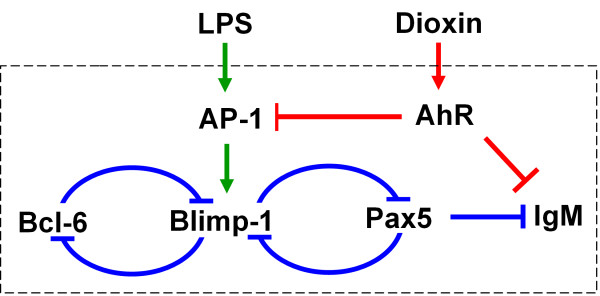
**Schematic illustration of the gene transcriptional program underlying B cell terminal differentiation and disruption by dioxin**. Consistent with the coupled mutual inhibitions among the transcriptional repressors Bcl-6, Blimp-1, and Pax5, B cells have a transcriptional profile of high Bcl-6, low Blimp-1, high Pax5, and low IgM. Plasma cells have the inverse transcriptional profile. Arrows indicate activation, and lines ending with a bar indicate inhibition.

Accurate evaluation of the immune health risk from exposure to dioxin-like compounds requires a mechanistic understanding of the biochemical network that underlies B cell differentiation and the manner in which these chemicals interfere with the operation of the network. As with many biochemical processes involved in cell fate decisions [10-13], the differentiation of B cells to plasma cells is mediated by a transcriptional program involving interacting transcription factors [1,14]. At the core of this gene regulatory network are two coupled double-negative feedback (mutual inhibition) loops among three transcriptional repressors: B cell lymphoma 6 (Bcl-6), B lymphocyte induced maturation protein 1 (Blimp-1), and paired box 5 (Pax5). Specifically, Blimp-1 is able to transcriptionally repress Bcl-6 and Pax5 [15-17]; and reciprocally, both Bcl-6 and Pax5 can repress Blimp-1 gene expression [18-20] (Figure 1). It is likely that the coupled double-negative feedback loops can form a bistable system, allowing cells to choose from one of two mutually exclusive and discrete states: naïve B cells or differentiated plasma cells [21]. Such a bistable system would provide a basis for the all-or-none differentiation observed with individual B cells [22-26]. Importantly, a bistable circuit would also make the differentiation physiologically irreversible, a property that is likely to contribute to the maintenance of the acquired immunity after antigen encounter.

Consistent with the idea of two mutually exclusive steady states for a bistable system, naïve B cells are characterized by a transcriptional profile of low Blimp-1 and high Bcl-6/Pax5 expression, whereas plasma cells feature the opposite profile: high Blimp-1 and low Bcl-6/Pax5 [1,27]. Pax5, acting as a transcriptional repressor, is essential for maintaining the B cell identity [28]. It actively suppresses transcription of the immunoglobulin molecular components including the heavy chain (IgH), κ light chain (Igκ), and J chain, as well as X box protein-1 (XBP-1) which promotes the formation of the cellular apparatus for immunoglobulin assembly and secretion [29]. The transcriptional repression of these genes by Pax5 ensures that naïve B cells neither produce nor secrete antibodies. Upon antigen encounter, the transcriptional profile in naïve B cells is reversed. High expression of Blimp-1, the 'master regulator' of B cell terminal differentiation, activates a number of downstream genes responsible for the plasma cell phenotype [16]. Most importantly, a simultaneous decrease in Pax5 gene expression releases IgH, Igκ, and J chain from repression, leading to enhanced production and secretion of immunoglobulin by plasma cells [28,29]. Depending on the type of antigen or B cell stimulus and whether T cells are involved, distinct signal transduction pathways may be activated to drive B cells to differentiate into plasma cells [30-33]. In the case of the bacterial endotoxin lipopolysaccharide (LPS), a polyclonal B cell activator, the differentiation is driven, at least in part, via AP-1, a dimeric transcription factor that upregulates Blimp-1 gene transcription [18,34].

The suppressive effect of TCDD and dioxin-like compounds on the humoral immune response is currently believed to occur via the following mode of action. By binding and activating the aryl hydrocarbon receptor (AhR), TCDD inhibits AP-1 gene transcription indirectly, leading to reduced AP-1 binding to the Blimp-1 promoter [35,36]. Since AP-1 mediates the action of LPS, TCDD would thus impair the capability of LPS to drive the switching of the transcriptional gene circuit that underlies B-to-plasma cell differentiation, thereby suppressing the formation of antibody-secreting plasma cells [6]. In addition, TCDD-activated AhR binds to the dioxin response element in the 3' α enhancer of the IgH gene, in a limited fashion inhibiting transcription of this immunoglobulin component [37].

Gene expression is inherently stochastic [38]. This is particularly true for low-abundance proteins, such as transcription factors, for which the steady-state expression level may fluctuate significantly [39-44]. In many circumstances, this intrinsic noise in protein expression is undesirable for cellular homeostasis and as such needs to be minimized. On the other hand there are instances where noise in protein expression is exploited by cells to generate advantageous non-genetic variability in cellular phenotype [12,45-47]. When an antigen or polyclonal B cell activator is encountered, the all-or-none differentiation of individual B cells occurs in a seemingly random fashion with new plasma cells appearing at various times [22,32,34]. This heterogeneity in the differentiation response is likely to be a key factor contributing to the dose response pattern for a population of B cells. It is possible that the intrinsically stochastic gene expression of Blimp-1, Bcl-6, and Pax5 is responsible for the observed heterogeneity in the differentiation response among otherwise identical B cells. In our previous work, we have suggested the emergence of bistability with a deterministic representation of the transcriptional network underlying B cell differentiation [21]. In the current paper, we use a stochastic computational model of this transcriptional network (Figure 1), constrained by experimental data, to examine how stochastic fluctuations in the abundance of the key transcriptional repressors can make B cell differentiation a dose-dependent probabilistic event. The present study indicates that the stochastic effect plays a central role in modulating the shape, especially the steepness, of the dose response curves for both LPS and TCDD, a result with significant implications for quantitative risk assessment of dioxin immunotoxicity.

## Methods

### Animals

Virus-free, female B6C3F1 mice (6 weeks of age) were purchased from Charles River (Portage, MI). Mice were randomized, transferred to plastic cages containing bedding (five per cage), and quarantined for 1 week. Mice were given food (Purina certified laboratory chow) and water *ad libitum *and were not used until their body weight was 17-20 g. Mice were used in accordance with the guidelines set forth by the Michigan State University Institutional Animal Care and Use Committee (East Lansing, MI).

### B cell isolation, activation, and TCDD addition

Spleens were isolated and made into single-cell suspensions aseptically. Primary B cells were isolated from the splenocytes by depleting all other cell types by magnetic separation using the B cell isolation kit from Miltenyi Biotec Inc. (Auburn, CA). B cell purity was always greater than 95% as assessed by flow cytometry. B cells were activated with different concentrations of LPS (Sigma-Aldrich Co., St. Louis, MO) at 3 × 10^6 ^cells/ml in RPMI 1640 supplemented with 10% BCS, 100 U/ml penicillin, 100 μg/ml streptomycin, and 50 μM 2-ME. Vehicle (0.02% DMSO) and appropriate TCDD concentrations (AccuStandard, New Haven, CT) were added just prior to LPS addition in a 48-well tissue culture plate.

### ELISpot assay

ELISpot assay was performed as previously described with a few modifications [48]. ELISpot filter plates (MultiScreen-HA) (Millipore, Billerica, MA) were precoated with goat anti-mouse IgM antibody (Sigma-Aldrich Co., St. Louis, MO) at 5 μg/ml in a sodium bicarbonate buffer (pH 9.6), washed and blocked with PBS containing 5% BSA. Purified B cells were washed twice and diluted in RPMI containing 10% BCS and loaded into the culture plate wells. Typically 1-4 × 10^3 ^cells in 100 μl cell culture media were incubated on precoated plates overnight at 37°C with 5% CO_2_. After incubation, plates were washed and biotin-conjugated goat anti-mouse IgM antibody (Sigma-Aldrich Co., St. Louis, MO) was added to the plates at 1 μg/ml and incubated for 2 h at room temperature. To develop spots, streptavidin-HRP was added (Sigma-Aldrich Co., St. Louis, MO) followed by the aminoethylcarbazole per staining kit instructions (Sigma-Aldrich Co., St. Louis, MO). Data was acquired and analyzed using the CTL ImmunoSpot system (Cellular Technology Ltd., Shaker Heights, OH).

### Measurement of IgM Secretion

IgM secretion was measured using an enzyme-linked immunesorbent assay protocol as previously described [49]. Briefly, supernatants were collected from experimental cultures from which LPS-induced IgM antibody responses were evaluated. Anti-mouse IgM capture antibody (Sigma-Aldrich Co., St. Louis, MO) was added to wells of a 96-well microtiter plate (100 μl/well at 5 μg/ml) and incubated at 4°C overnight. The plate was washed three times with 0.05% Tween-20 in PBS and three times with dH_2_O. Blocking buffer (3% BSA in PBS) was added to each well and incubated at room temperature for at least 1.5 h. This was followed by the same washing steps described above. Standards (mouse IgM) or supernatant samples were then added to the blocked plate and then incubated at 37°C for 1.5 h. After the incubation, the plate was washed again, followed by addition of 100 μl HRP-conjugated goat anti-mouse IgM detection antibody. After 1.5 h at 37°C, the plate was again washed and 100 μl ABTS (1 mg/ml; Roche, Indianapolis, IN) was added. The detection of the HRP substrate reaction was conducted over a 1 h period using a Synergy HT automated Microplate reader with a 405-nm filter (Bio-Tek, Winooski, VT). The KC4 computer analysis program (Bio-Tek, Winooski, VT) calculated the concentration of IgM in each well based on a standard curve generated from the absorbance readings of known IgM concentrations.

### Model structure

The model structure is illustrated in Additional File 1: Figure S1. Simulation of gene expression control of the transcriptional repressors Bcl-6, Blimp-1, and Pax5 was based on the current understanding of eukaryotic gene regulation [43,44,50,51]. At any given time, a gene could be in one of two discrete transcriptional states: inactive or active, corresponding to the compact and relaxed chromatin structure of the promoter, respectively. Once in the active state (GENE1, Additional File 1: Figure S1), the gene is transcribed at a relatively constant rate; in the inactive state (GENE0), no transcription occurs. Transitions between the inactive and active states (i.e., gene activation and deactivation) are controlled by transcriptional activators and repressors specifically targeting the promoter. In addition to promoter transition, subsequent steps including transcription, translation, and mRNA and protein degradation were modeled for each of the three transcriptional repressors. Since these key steps contribute, to varying degrees, to the stochastic fluctuation of gene expression [38-44], their explicit inclusion adds variability to the switching behavior of the bistable circuit. In turn, the stochastic behavior of the bistable switch likely forms the basis for the heterogeneous differentiation response observed for a population of B cells stimulated with an antigen or B cell activator [22,32,34].

The negative mutual regulation among Bcl-6, Blimp-1, and Pax5 is modeled based on the modes of repression reported in the literature. Blimp-1 represses gene expression of both Bcl-6 and Pax5 [15-17]. As a transcriptional repressor, Blimp-1 suppresses target genes by recruiting co-repressors such as histone deacetylase, histone methyltranferase, and those belonging to the Groucho family [52-54]. Functioning as chromatin-modifying enzymes, these co-repressors alter the local chromatin structure to a compact (transcriptionally inactive) state. Based on this *active *mode of repression, the transcriptional repression of Bcl-6 and Pax5 by Blimp-1 was implemented by having Blimp-1 promote the deactivation step of these two genes. Pax5 in turn represses Blimp-1 gene expression directly [20], also by an active mechanism mediated through recruitment of co-repressors from the Groucho family [55,56]. Therefore, repression of Blimp-1 by Pax5 was implemented by having Pax5 promote the deactivation step of Blimp-1 gene. Although the transcriptional repressor Bcl-6 may directly bind to target genes, it exerts its effect on Blimp-1 indirectly through AP-1. By binding and sequestering AP-1, Bcl-6 blocks its transcriptional activity, thus functioning as a *passive *repressor [18]. Since AP-1 positively regulates Blimp-1 gene expression, repression of Blimp-1 by Bcl-6 was implemented by having Bcl-6 impinge upon the activation step of Blimp-1 gene, thus curbing the maximal induction of Blimp-1 by AP-1. Through these specific transcriptional regulations, a gene circuit is established in the form of coupled double-negative feedback loops among Bcl-6, Blimp-1, and Pax5 (Additional File 1: Figure S1).

Activation of the transcriptional program underlying B cell terminal differentiation occurs in a stimulus-dependent manner, involving various cytokines and cell surface receptors [14]. In the case of LPS, binding to its cognate Toll-like receptor 4 (TLR4) initiates a sequence of intermediate intracellular signaling events, culminating in the activation of the AP-1 protein complex [34,35]. For simplicity, the activation of AP-1 by LPS-bound TLR4 was modeled as a single phosphorylation event. Like many other membrane-residing receptors, TLR4 is usually downregulated after occupied by LPS, possibly through a desensitization mechanism that includes receptor internalization [57,58]. Downregulation of TLR4 was implemented here by ascribing a higher rate constant to the turnover of the LPS-bound receptor compared to that of the free receptor. Inclusion of TLR4 downregulation enables the model to recapitulate the attenuation of AP-1 signaling in time following its initial activation, as observed in B cells stimulated with LPS and other cytokines [34,35].

The suppression of AP-1 activity by TCDD in LPS-activated B cells was assumed to occur in an AhR-dependent manner. Although the precise mechanism for this process is unknown, a decrease in the AP-1 subunit c-jun was observed in the AP-1 DNA-binding complex in the presence of TCDD [35]. For simplicity, AP-1 was treated as a single entity in our model with its synthesis rate negatively regulated by TCDD. In the absence of detailed information at the molecular level on the signaling pathways by which TCDD-AhR affects AP-1, the specific regulation of AP-1 by TCDD-AhR was described in the equation as a continuous function of the concentration of TCDD-AhR. Immunoglobulin M (IgM), which consists of several subunits, was also modeled as a single entity subject to negative control by both Pax5 and AhR. A first-order secretion rate was assumed for intracellular IgM, with the rate constant large enough that intracellular degradation of IgM is negligible.

### Model implementation and modeling tools

Reaction details including ordinary differential equations, parameter values, and initial steady-state conditions are provided in Additional File 1: Tables S1 - S3. Unless otherwise indicated, the unit of abundance used for state variables in the model is number of molecules per cell. The deterministic version of the model was implemented in PathwayLab (InNetics Inc., Linköping, Sweden), while the stochastic version was implemented in the BioNetS program [59], based on Gillespie's stochastic simulation algorithm [60]. Both versions of the model were exported into MatLab (The Mathworks, Inc., Natick, MA, USA) for analysis and simulation. The model in SBML format is provided in Additional File 2. Bifurcation diagrams were generated using the XPP-AUT program [61]. The effective Hill coefficients (n_H_) of simulation-generated dose response curves were estimated by using the equation n_H _= ln81/ln(X_90_/X_10_), where X_90 _and X_10 _are the doses that produce 90% and 10% of the maximal response, respectively.

## Results

### Bistability of the coupled double-negative feedback loops

**Figure 2:**
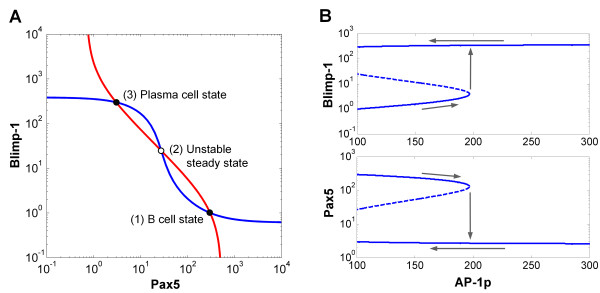
**Analysis of bistability in the gene regulatory network underlying B cell terminal differentiation (see Additional File **1**: Supplementary Material for model details)**. **(A) **Steady-state stimulus-response curves between Blimp-1 and Pax5 under zero LPS and TCDD. The red curve was obtained with Blimp-1 level as the independent variable (i.e., stimulus) and the steady-state level of Pax5 as the response; the blue curve was obtained with Pax5 level as the independent variable and the steady-state level of Blimp-1 as the response. The blue curve represents an ultrasensitive response of Blimp-1 to Pax5 due to the presence of the Bcl-6/Blimp-1 double-negative feedback loop. The ultrasensitivity enables the blue curve to intersect with the red curve at three points, indicative of bistability. Intersection point 1 represents the stable B cell state; intersection point 3 represents the stable plasma cell state; and intersection point 2 is an unstable steady state. **(B) **Bifurcation analysis using AP-1p (active form of AP-1) as the independent parameter. Vertical arrows indicate that there is a deterministic threshold value of AP-1p required to turn on the bistable switch. Leftward arrows indicate that the bistable switch is irreversible even when AP-1p drops back to the basal level of 100.

The coupled feedback loops comprising Bcl-6, Blimp-1, and Pax5 exhibit bistability, a desirable systems property for cells undergoing differentiation [62]. The undifferentiated and differentiated cellular states are unambiguously separated, relatively stable, and the transition from the former to latter state is physiologically irreversible (Figure 2). The bistable property of a feedback system can be graphically analyzed by the reciprocal open-loop steady-state stimulus-response relationships between any pair of variables in the feedback loops [63]. Here we describe such stimulus-response relationships in the multivariable model as "null curves" for their correspondence to nullclines in a two-variable system. Null curves were obtained for the Blimp-1 and Pax5 variable pair (Figure 2A) at the unperturbed condition (LPS = 0 and TCDD = 0). The two null curves intersect three times, indicating three possible steady states at which the system can settle. Intersection points 1 and 3 are stable steady states, representing the resting mature B cell (low Blimp-1 and high Pax5) and plasma cell (high Blimp-1 and low Pax5) states, respectively. Intersection point 2, situated between the two stable states, represents an unstable steady state. These three steady states are essentially projections onto the Blimp-1-Pax5 plane of the attainable fixed points of the system in multi-dimensional phase space. In that phase space, an imaginary boundary passing through point 2 (corresponding to the "separatrix" for a two-variable system) divides the phase space into two basins of attraction [64]. On one side of the boundary, the system tends to converge to the B cell state; on the other side, it converges to the plasma cell state.

By activating AP-1, LPS triggers the differentiation of B cells to plasma cells. Bifurcation analysis (Figure 2B) indicates that as the steady-state AP-1p (active form of AP-1) level increases (rightward arrows), Blimp-1 increases and Pax5 decreases initially by small amounts. However, once AP-1p reaches a threshold value of about 200 molecules, the B cell switches in a discrete fashion to a plasma cell state (vertical arrows). This abrupt transition underlies an all-or-none, switch-like response and signifies a true discontinuity that precludes the cell from settling at an intermediate state. The switch also exhibits hysteresis. Starting from the plasma cell state, as AP-1p is reduced (leftward arrows) to its basal level (~100), the system remains in the differentiated plasma cell state with high Blimp-1 and low Pax5/Bcl-6 levels. In the context of humoral immune response, this irreversibility of the switch ensures that the antibody-secreting plasma cell phenotype persists after the initial antigen stimulus recedes, thus serving as a maintenance mechanism for the acquired immunity.

### Stochastic response of B cells to LPS stimulation

In a deterministic model of a bistable system, the occurrence of switching from one state to the other and the time when it occurs are uniquely determined by the level of the input stimulus [21]. If the biochemical circuits are nearly identical across a population of B cells, the individual responses of these cells would be expected to be similar. However, experimental observations indicate that the responses of individual cells to a given activator are rather heterogeneous [22,32,34], even in an isogenic B cell population [65]. Some cells differentiate into antibody-secreting plasma cells at early times, some do so at late times, and many cells remain undifferentiated in the time window of observation. Thus the number of plasma cells that appear after B cell activation is time-dependent. This heterogeneous response pattern is likely to be important in shaping dose response behaviors, which are quantitative measurements necessary for evaluating the health risk of immune-suppressive chemicals. Therefore a more explicit description of the heterogeneous response of individual B cells is needed than is provided by a deterministic model.

**Figure 3:**
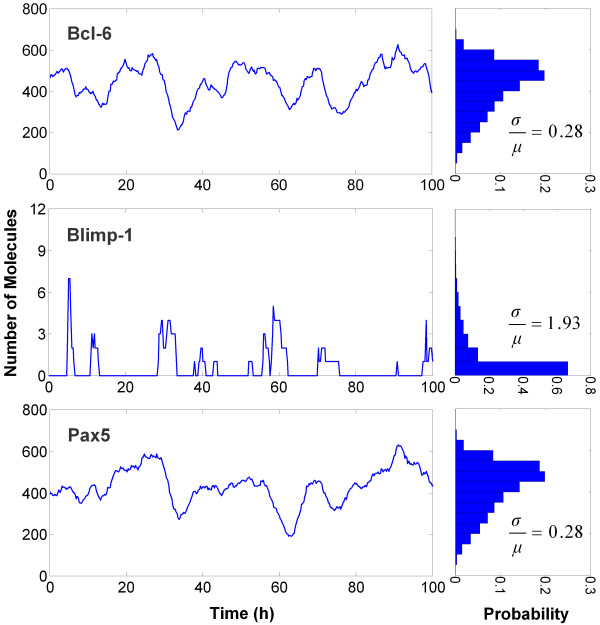
**Simulated stochastic gene expression of Bcl-6, Blimp-1, and Pax5 in the B cell state**. The histograms (right panels) generated from a population of 10^5 ^simulated cells illustrate the degree of variability in protein abundance (noise). The noise level is quantified by the coefficient of variation, σ/μ (where σ is the standard deviation and μ the mean). Due to the low abundance in B cells, Blimp-1 expression is much more noisy (larger σ/μ) than Bcl-6 and Pax5, and fluctuates in a pulsatile manner. The stochastic, pulsatile expression of Blimp-1 is primarily responsible for the heterogeneous switching response of individual B cells to LPS stimulation.

Heterogeneity in the occurrence and timing of individual B cell differentiation is likely to result from stochastic fluctuations in gene expression of the key transcription factor Bcl-6, Pax5, and in particular, Blimp-1 - as discussed below. We have simulated this scenario using the BioNetS program [59] based on Gillespie's stochastic algorithm [60]. Since Blimp-1 is expressed at a low level in the B cell [66], it exhibits a pulsatile expression pattern and is much more "noisy" than Bcl-6 or Pax5 (Figure 3, left panels). The coefficient of variation, a measure of the noise level, of Blimp-1 is almost seven times greater than that of Bcl-6 and Pax5 (Figure 3, right panels). Most of the noise in Bcl-6 and Pax5 protein expression actually originates from Blimp-1. This can be demonstrated by replacing the stochastic input from Blimp-1 to Bcl-6 and Pax5 genes with a constant equal to the mean Blimp-1 level in the B cell state, whereby the noise in expression of Bcl-6 and Pax5 is significantly reduced (Additional File 1: Figure S2).

**Figure 4:**
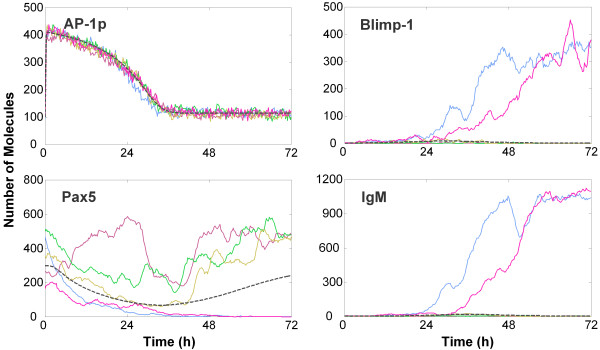
**Heterogeneous responses of stochastically simulated B cells under continuous stimulation with 10 μg/ml LPS for 72 h**. Of the five simulated B cells, two cells clearly turn on the bistable switch, becoming IgM-secreting plasma cells; the other three remain unresponsive. Dashed gray lines are trajectories from deterministic simulations, indicating that the bistable circuit is not switched on with the same set of parameter values in a deterministic model.

Despite the noisy expression of Blimp-1, Bcl-6 and Pax5, the B cell state is very stable: in the absence of LPS, only a negligible fraction of B cells (about 0.02%) switch spontaneously to the plasma cell state in 200 hours. However, the stochastic fluctuations in gene expression, particularly of Blimp-1, are sufficiently high that the switching behavior of the bistable gene circuit in response to LPS stimulation becomes probabilistic and exhibits substantial cell-to-cell variability. For example, among five stochastically simulated B cells tracked over a period of 72 h under 10 μg/ml LPS (Figure 4, solid lines), two became activated at different time points, as reflected in the decrease in Bcl-6 (data not shown) and Pax5 levels, and increase in Blimp-1 and IgM protein levels. The other three cells remained unresponsive, thus reproducing the expected all-or-none differentiation response of individual B cells. In contrast to the divergent switching behavior of the core bistable gene circuit itself, activation of the upstream AP-1 protein by LPS follows a similar pattern in deterministic and stochastic simulations - trajectories for the five stochastically simulated B cells closely clustered around the deterministic result (Figure 4, top left panel). The desensitization of TLR4 through internalization (as discussed in the Methods section) causes the rise in AP-1p level to be transient. Interestingly, the deterministic implementation of the circuit does not turn on the bistable switch at all (Figure 4, dashed lines: Blimp-1 and IgM levels remain low), even with saturating LPS concentrations. This outcome indicates that while AP-1p may transiently exceed the threshold value (~200, see Figure 2B), it does not persist above that level long enough to activate the bistable switch deterministically. Nonetheless, the same parameters are sufficient to cause a significant number of B cells to switch into plasma cells in a noisy gene expression environment.

**Figure 5:**
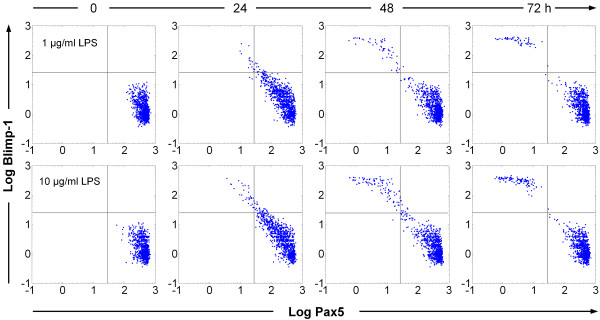
**Stochastic simulations indicating all-or-none, but heterogeneous responses in individual B cells using Blimp-1 and Pax5 as dual expression markers**. The B cell population (1000 cells) splits into two distinct clusters under continuous LPS stimulation for 72 h - the cluster with low Blimp-1/high Pax5 represents B cells, while the cluster with high Blimp-1/low Pax5 represents plasma cells. Individual B cells switch to the plasma cell state randomly over time and higher LPS concentration results in more plasma cells. Top panels: 1 μg/ml LPS, bottom panels: 10 μg/ml LPS. Note: to help visualize cells with zero copy number of Blimp-1 or Pax5 proteins on logarithmic scale, a number drawn randomly from a log-normal distribution (with mean = 1 and variance = 0.1) was added to the simulated protein expression data. This in effect mimics background fluorescence in flow cytometry studies measuring protein expression.

**Figure 6:**
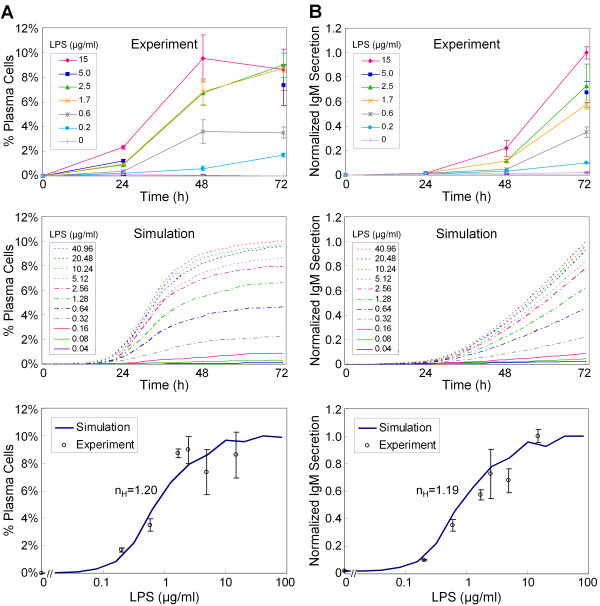
**Comparison of experimental and stochastic simulation results for percentage of plasma cell formation (A) and accumulated IgM secretion *(B) *under continuous LPS stimulation for 72 h**. The simulated LPS dose response curves obtained at 72 h for percentage plasma cells (A, bottom panel) and IgM secretion (B, bottom panel) have an estimated Hill coefficient of 1.20 and 1.19, respectively. A simulated cell is counted as a plasma cell if the IgM level in the cell is above 200. The same standard was used for results shown in other figures. Accumulated IgM secretion data were normalized to the maximum value at 72 h for comparison. *In vitro *experimental results were from triplicate samples of primary mouse B cells for each LPS concentration, while simulation results were obtained from 10^4 ^stochastically simulated cells for each LPS concentration.

The effect of probabilistic switching in a population of B cells under LPS stimulation is better visualized by simultaneous monitoring of Blimp-1 and Pax5 gene expression in our stochastic simulations, analogous to experiments using dual-fluorescence flow cytometry (Figure 5). These simulations produced largely two distinct clusters of data points, representing naïve B cell (low Blimp-1 and high Pax5) and plasma cell (high Blimp-1 and low Pax5) populations, respectively. The data points located in between these two clusters (particularly at 24 and 48 h) represent the small number of cells still in the process of switching from the B cell to the plasma cell state. The plasma cell cluster gradually grows in size over a period of 72 h, representing heterogeneous responses among a population of individual B cells. A higher LPS dose results in higher plasma cell formation at each time point (Figure 5, lower panels). The dynamics of these time- and dose-dependent changes in the percentage of plasma cell formation is qualitatively similar to the experimental results observed with primary mouse B cells (Figure 6A, top and middle panels). In the first 24 h, the response is relatively small, due likely to the time required for completion of the switching process. The largest increase in the plasma cell population occurs between 24-48 h, before slowing dramatically between 48-72 h, a result related to the decline in AP-1p levels (Figure 4). The simulated dose response for percentage plasma cell formation at 72 h adequately recapitulates the experimental observations (Figure 6A, bottom panel). The curve, with an estimated apparent Hill coefficient of 1.20, does not appear to have abrupt changes, suggesting that the number of plasma cells formed is largely a graded function of the LPS dose. Experimental measurement and computer simulation both show that accumulated IgM secretion accelerates over the entire period of observation (Figure 6B, top and middle panels). As with percentage of plasma cell formation, the predicted response for IgM secretion at 72 h also appears to be a graded function of LPS dose (Figure 6B, bottom panel). The estimated Hill coefficient is 1.19, similar to that for plasma cell formation. A more detailed examination of the low-dose effect of LPS further confirms that regardless of the endpoint response examined, the B cell population as a whole would respond in a continuous, graded fashion to increasing doses of LPS (Additional File 1: Figure S3), even as the switching of individual cells is all-or-none.

**Figure 7:**
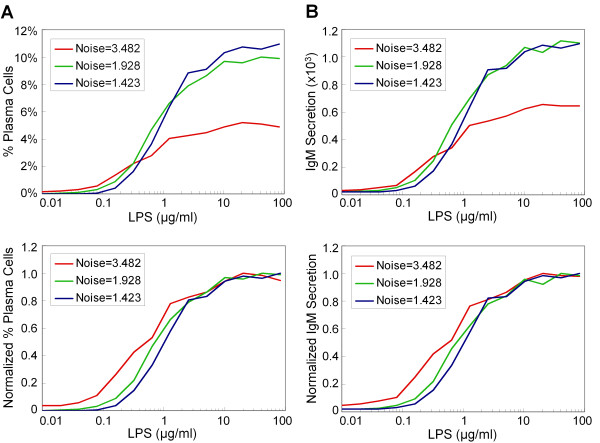
**Simulated effects of stochastic noise in Blimp-1 gene expression on the LPS dose response curves for (A) percentage of plasma cell formation and (B) averaged accumulated IgM secretion per cell**. The dose response curves were obtained from 10^4 ^simulated cells under continuous LPS stimulation for 72 h. Blimp-1 noise level was decreased (increased) by simultaneously increasing (decreasing) Blimp-1 transcription rate constant (k13) and decreasing (increasing) Blimp-1 translation rate constant (k15) by the same 5-fold from default values, an alteration that kept the deterministic steady-state level of Blimp-1 protein unchanged. The estimated Hill coefficient and ED_50 _values are shown in Table 1.

To examine how the level of noise in gene expression affects the shape and steepness of the dose response curves, we tuned the noise level of Blimp-1 in the model. This was done by simultaneously altering the transcription and translation rate constants of Blimp-1 by similar magnitudes, but in opposite directions, such that the deterministic steady-state level of Blimp-1 protein remained the same [39]. In addition to suppressing the maximal response for percentage of plasma cell formation and IgM secretion (Figure 7, top panels), increasing noise level in Blimp-1 gene expression tends to decrease the steepness, as illustrated by the normalized responses (Figure 7, bottom panels), and consequently the Hill coefficient (Table 1) of the dose response curves for LPS. Decreasing the noise level has the opposite effect. In addition, a higher degree of stochastic fluctuation in Blimp-1 protein level also shifts the dose response curves to the left with a decreasing ED_50 _value (Figure 7, bottom panels; also see Table 1).

**Table 1:** Effects of noise in Blimp-1 protein expression on the steepness and position of the LPS dose response curves.

	% plasma Cells		IgM Secretion	
	
Blimp-1 noise	n_H_	ED_50_	n_H_	ED_50_
1.423	1.253	1.074	1.260	1.100
1.928	1.201	0.755	1.188	0.843
3.482	0.959	0.570	1.023	0.650

Note: The quantitative effects of altering Blimp-1 noise level were evaluated for the dose response curves of percentage of plasma cell formation and IgM secretion presented in Figure 7. Blimp-1 noise level is expressed as the coefficient of variation of Blimp-1 protein abundance. The default parameter values of the model generate a coefficient of variation of 1.928. The lower (higher) noise level of 1.423 (3.482) was obtained by increasing (decreasing) Blimp-1 transcription rate constant (k13) and decreasing (increasing) Blimp-1 translation rate constant (k15) by 5-fold, which kept the deterministic steady-state level of Blimp-1 protein unchanged. n_H_, Hill coefficient; ED_50_, concentration of LPS (μg/ml) producing 50% of the maximum response.

### Disruption of the bistable switch by TCDD

**Figure 8:**
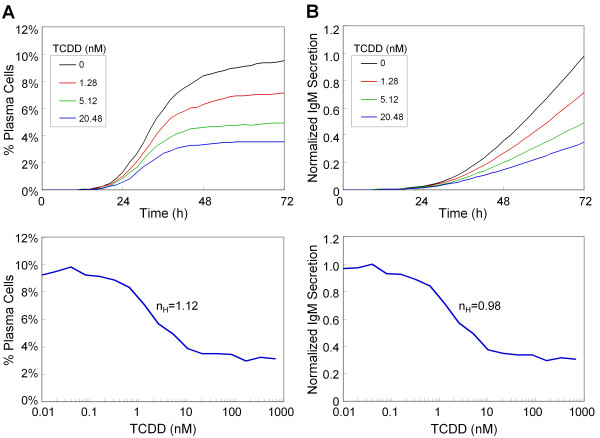
**Simulated suppressive effect of TCDD on LPS-stimulated (A) percentage of plasma cell formation and (B) averaged accumulated IgM secretion per cell**. The results were obtained from 10^4 ^simulated cells under continuous LPS (15 μg/ml) and TCDD treatment for 72 h. The dose response curves with respect to TCDD obtained at 72 h for percentage plasma cells (A, bottom panel) and IgM secretion (B, bottom panel) have an estimated Hill coefficient of 1.12 and 0.98, respectively.

**Figure 9:**
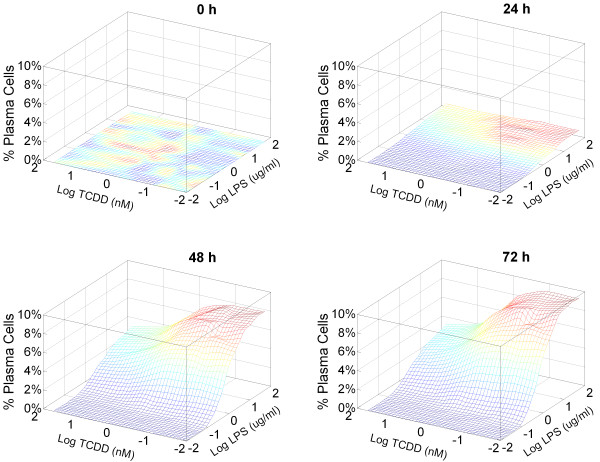
**Simulated dose response surfaces for percentage of plasma cell formation at 0, 24, 48, and 72 h time points under continuous treatment with various combinations of LPS and TCDD concentrations**. The results were from 10^4 ^simulated cells for each concentration combination.

Acting via AhR, TCDD and dioxin-like compounds disrupt B cell terminal differentiation and immunoglobulin production by repressing gene transcription of the AP-1 protein and components of IgM [35-37]. Simulations of the B cell transcription network show that TCDD inhibits, in a dose-dependent manner, both the number of plasma cells formed and aggregated IgM secretion in response to LPS stimulation (Figure 8, top panels). The maximal suppression is about 30% of the control level, comparable to that observed experimentally with primary B cells (Additional File 1: Figure S4) and consistent with earlier reports [6,67,68]. As with LPS activation, the TCDD dose response curves also appear to be graded, with Hill coefficients of 1.12 and 0.98 for percentage of plasma cell formation and IgM secretion, respectively (Figure 8, bottom panels; and Additional File 1: Figure S5 for low concentrations of TCDD). In the network described here, the suppressive effect of TCDD on the probability of B cell differentiation arises from attenuation by TCDD of the LPS-induced transient activation of AP-1 (Additional File 1: Figure S6). As previously discussed, this step serves as the trigger switching the bistable gene circuit from the B cell to the plasma cell state. To further characterize the suppressive effects of TCDD, various combinations of LPS and TCDD concentrations were applied to the stochastic model. The simulated response surfaces at 48 and 72 h appear to exhibit gradual changes with respect to both LPS and TCDD concentrations (Figure 9), devoid of the precipitous transitions expected for a bistable system described with a set of deterministic equations.

**Figure 10:**
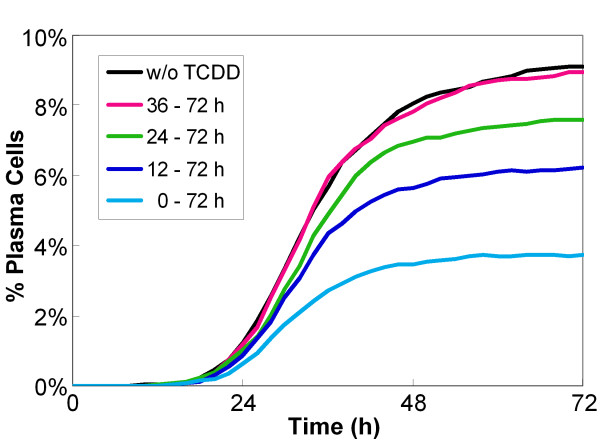
**Simulated effect of the timing of TCDD treatment on LPS-stimulated B cell differentiation**. The results were from 10^4 ^simulated cells with LPS at 10 μg/ml and TCDD at 10 nM. LPS treatment was continuous from 0 to 72 h. TCDD treatment started at different times as indicated and continued till 72 h.

Despite the fact that most plasma cells do not appear until about 24 h after the onset of LPS stimulation (Figure 6A), previous *in vitro *studies have demonstrated that effective suppression of plasma cell formation by TCDD requires that TCDD be present in the first 24 h following antigen challenge [6,69]. Simulations of the modeled network produce similar behaviors. As the onset of TCDD treatment is progressively delayed relative to LPS stimulation, the TCDD-induced suppression of plasma cell formation is gradually diminished, and completely lost for delays greater than 36 h (Figure 10). Although the molecular basis for this time-dependent suppression is not fully understood, the transience of antigen-stimulated AP-1 activation in our B-cell differentiation network is the key component responsible for the limited window of susceptibility to TCDD suppression. The desensitization of TLR4 after LPS binding in our model causes the AP-1p level to rise transiently, which then declines by 24 h and nearly returns to the baseline by 36 h (Figure 4). Were TCDD treatment started between 24 and 36 h, the time period of most effective suppression of LPS-induced AP-1 activation (i.e., 0-24 h) would be missed. B cells that have already committed to turning on the bistable switch within the first 24 h are irreversibly on the way to the plasma cell state and would not be affected by TCDD added at a later time.

**Figure 11:**
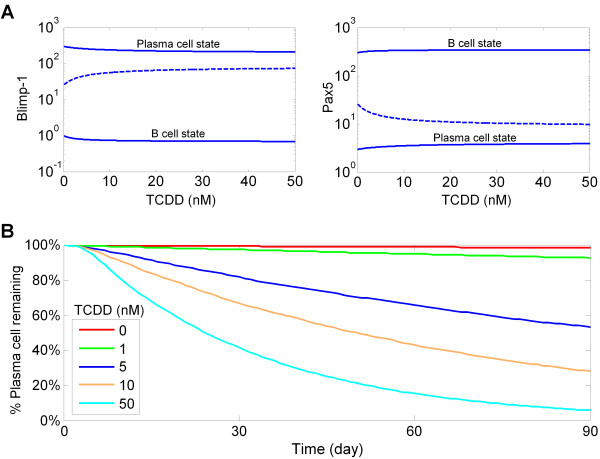
**Simulated effect of TCDD in destabilizing the plasma cell state**. **(A) **Bifurcation analysis in the absence of LPS indicates that as the concentration of TCDD increases, the plasma cell state (upper branch of the Blimp-1 vs. TCDD bifurcation diagram and lower branch of the Pax5 vs. TCDD bifurcation diagram) move closer to the intermediate unstable steady state (dashed branches), although they never coalesce. **(B) **Stochastic simulation using 10^4 ^plasma cells demonstrated that TCDD destabilized the plasma cell state over time in a dose-dependent manner.

As with many cell differentiation events, the differentiation of B cells to antibody-secreting plasma cells is believed to be a physiologically irreversible process. Our analysis suggests that the hysteresis inherent in the bistable switch comprising Bcl-6, Blimp-1, and Pax5 (Figure 2B) is responsible for this irreversibility. A bifurcation analysis in the absence of LPS reveals that as the concentration of TCDD is increased (Figure 11A), the plasma cell state (represented by the upper branch of the Blimp-1 vs. TCDD bifurcation diagram and lower branch of the Pax5 vs. TCDD bifurcation diagram) moves closer to the intermediate unstable steady state (dashed branches), although the two states never coalesce for high TCDD concentrations. This behavior suggests that the stable plasma cell state approaches the boundary in phase space that separates the basin of attraction of the B cell state from that of the plasma cell state. There is thus an increased probability that in the presence of TCDD, the stochastic fluctuations in the levels of key transcriptional repressors, especially those expressed at low levels in the plasma cell (i.e., Pax5 and Bcl-6), may swing the system across the fate-dividing boundary to the B cell state. This suggests that TCDD could disrupt the stability of the plasma cell state, making the B-to-plasma cell differentiation process reversible. In agreement with this prediction, stochastic simulations indicate that for a population of plasma cells subject to continuous presence of TCDD, the number of plasma cells decreases over time in a seemingly exponential fashion with the rate of reversion increasing with TCDD concentrations (Figure 11B).

## Discussion and Conclusions

Many cellular processes in living organisms, including sex determination, lineage specification, cell differentiation, proliferation, and apoptosis, are irreversible, all-or-none (binary) phenomena. It is increasingly recognized that such binary cellular processes are controlled by bistable biochemical circuits, which ensure the discreteness and irreversibility of these processes [62,70]. Most cellular bistable systems are based on molecular circuits of mutual activation and/or mutual inhibition between genes and proteins [71]. These molecular circuits often involve a number of proteins forming multiple feedback loops, as observed in systems for cell cycle control, oocyte maturation in *Xenopus laevis*, cell lineage specification and differentiation, etc [11,72-75]. The coupling of multiple feedback loops is believed to provide redundancy as well as robustness to the bistable property of the entire circuit [73,76]. In the B cell transcriptional network, double negative feedback loops between Bcl-6, Blimp-1, and Pax5 are coupled at the Blimp-1 node. The resulting bistability produces two mutually exclusive transcriptional profiles: (1) high Bcl-6/Pax5 and low Blimp-1, representing the B cell state; and (2) low Bcl-6/Pax5 and high Blimp-1, representing the plasma cell state (Figure 2A). This mutual exclusivity of stable gene expression patterns ensures definitive separation between two discrete cellular phenotypes and an all-or-none response in individual B cells to antigen stimulation. Further, once a B cell differentiates into a plasma cell, the plasma cell phenotype is retained even if the stimulating antigen is removed. This irreversibility is underpinned by hysteresis, another important biological property conferred by the bistable system (Figure 2B).

The switching from the B cell to plasma cell state is stimulated by specific antigens or polyclonal activators. In the case of LPS, the switching is mediated by alterations in concentration of active AP-1 protein (AP-1p) and characterized by a threshold concentration of AP-1p (Figure 2B). This in turn suggests a threshold for LPS, the initiating stimulus, and a switch-like dose response in a deterministic setting. In reality, the bistable gene circuit in the B cell has to operate in an inherently stochastic environment within the nucleus, which is likely to produce significant cell-to-cell variability. Our experimental data shows that individual B cells differentiate into plasma cells in a rather heterogeneous manner, with the percentage of plasma cells formed appearing to be a graded function of the LPS dose, devoid of the sharp transitions expected for an all-or-none bistable switch (Figure 6A). By considering the stochasticity in gene expression, our simulations recapitulate these observations. Thus whether a particular B cell will respond or not within a given time window of LPS stimulation, as well as the timing of the response, are both chance events resulting from intrinsically stochastic gene expression.

Due to the low expression level of Blimp-1 in B cells [66], its abundance is likely to show considerable moment-to-moment and cell-to-cell variability. In our implementation of the B-cell differentiation network, Blimp-1 fluctuates in a nearly pulsatile manner (Figure 3), which is consistent with the phenomenon of pulsatile mRNA production and bursting of protein translation observed for genes with low expression [77-80]. In the phase space of the bistable system, mature B cells would reside on one side of an imaginary boundary separating the two basins of attraction for the B cell and plasma cell states. Even in the absence of LPS, an occasional pulse in Blimp-1 expression may be large enough to send the system across the boundary to the plasma cell state. This might explain the spontaneous appearance of a negligibly small fraction of plasma cells observed in a population of B cells *in vitro *in the absence of any antigen [22,23,34,81]. Exposure to LPS, which activates the AP-1 protein, leads to increased Blimp-1 gene transcription. Due to the stochastic nature of Blimp-1 gene expression, the LPS-induced increase in Blimp-1 abundance takes the form of more frequent and larger Blimp-1 pulses (Additional File 1: Figure S7). These pulses increase the probability of the system crossing the fate-separating boundary and then being attracted to the plasma cell state eventually. Our analysis suggests that the cell-to-cell variability in Blimp-1 pulses at a given moment explains the heterogeneous switching behavior of individual B cells. Previous computational studies have shown that stochastic fluctuations could obscure the threshold of the switching behavior of a bistable system, making the dose response less switch-like [82,83]. In our implementation of the B cell differentiation network, sufficiently large stochastic fluctuations in Blimp-1 expression result in substantially graded response for percentage plasma cell formation with an estimated Hill coefficient close to 1. This number, a measure of the steepness of the dose response curve, is inversely correlated with the level of noise in Blimp-1 protein expression (Figure 7 and Table 1).

In summary, the bistable gene circuit operating in a stochastic environment in B cells confers two essential properties to the humoral immune response. First, bistability ensures that distinct, mutually exclusive phenotypes are associated with the B cell and plasma cell, and that the switching from the B cell to plasma cell phenotype is irreversible so as to maintain the acquired immunity for a period of time following antigen encounter. Second, stochastic fluctuations in protein expression provide the necessary variability for the differentiation response to becomes probabilistic. This allows the number of plasma cells formed and the total amount of antibody produced to scale in a graded manner with the antigen dose. Numerous studies have shown that noise in protein expression can be exploited by cells to generate necessary non-genetic variability in cellular phenotype and fate [12,45-47,84-86]. For example, intrinsic noise in ComK protein expression in the soil bacterium *Bacillus subtilis *allows a small fraction of cells to reside in the competent state capable of DNA uptake rather than in a vegetative state. This strategy increases fitness of the species by increasing phenotypic diversity among a population of genetically identical bacteria [12,87]. A recent study by Spencer et al. demonstrated that protein expression noise may be responsible for the variability in apoptotic response observed in clonal populations of mammalian cells [45]. It is thus likely that noise in gene expression is utilized by B cells to launch a humoral immune response of appropriate magnitude (as measured by the extent of plasma cell formation and aggregated IgM secretion) in handling the amount of pathogen in the body. This proposed effect of variability at the molecular level on the overall dose response curves is similar to the hypothesis on chemical carcinogenesis at the human population level proposed by Lutz, which states that even if each human individual had a specific response threshold for a carcinogen, variability among individuals would result in a continuous dose response curve for a human population [88].

The molecular basis of the immunosuppressive effect of the environmental contaminant TCDD and similar compounds acting through AhR remains incompletely understood at the molecular level. A primary goal in developing cell-based quantitative network simulations of toxic response is to evaluate the shape of the dose response curve over a broad range of exposures. To this end, it is necessary to acquire mechanistic understanding of how dioxin-like compounds interact with the gene transcriptional network underlying the B cell terminal differentiation program. As we have tried to demonstrate in the current work, computational modeling approaches can be helpful in attaining this goal. Through activation of the AhR signaling pathway, TCDD, a potent dioxin compound, suppresses AP-1 signaling, a key mediator of B cell differentiation [35,36]. This attenuation of antigen-stimulated AP-1 activation by TCDD (Additional File 1: Figure S6) makes it more difficult for a B cell to switch to the plasma cell state. In a stochastic gene expression context, this inhibitory effect of TCDD is manifested as a reduction in the probability of B cell differentiation. With fewer antibody-secreting plasma cells formed, the humoral immunity is compromised. Importantly, stochasticity in gene expression of transcription factors transforms the binary, all-or-none response occurring in individual B cells into a much more graded response to TCDD for a population of B cells (Figure 8). Our computational model, which incorporates as default a continuous description of TCDD affecting AP-1 and simulates *in vitro *experimental scenarios, suggests that the graded nature of the suppressive effect on B cell differentiation may extend well into the low-dose region, where no abrupt transitions were observed (Additional File 1: Figure S5).

Differentiation of mature B cells into plasma cells is a terminal, physiologically irreversible process. Some of the newly differentiated plasma cells migrate to the bone marrow, where, aided by survival signals from stromal cells, they can survive for several months [89-91]. Independent of memory B cells, these long-lived plasma cells contribute to acquired immunity against pathogens by continuing to secrete antibody molecules for an extended period of time. While the effect on B cell differentiation has been a focus of research concerning immunotoxicity of TCDD and related compounds, its possible effect on the fate of terminally differentiated plasma cells has not received much attention. In its present form, our B cell response network suggests that TCDD may alter the phase space landscape of the transcription program, bringing the plasma cell state closer to the boundary separating it from the B cell state (Figure 11A). This would make it easier for stochastic fluctuations to switch plasma cells back to the B cell state. This behavior occurs in the stochastic implementation but not in the deterministic mode with an identical parameter set. Stochastic simulations suggest that TCDD destabilizes plasma cells in a dose-dependent manner, possibly allowing these cells to dedifferentiate back to a B cell phenotype (Figure 11B). While the predicted decline in the number of plasma cells due to phenotype reversal is a slow process, the impairment to the acquired humoral immunity could be tangible, given that long-lived plasma cells may survive in the bone marrow for up to several months [89-91].

Although cell differentiation is traditionally regarded as a physiologically irreversible process, gene manipulation in terminally differentiated cells has been shown to successfully reprogram these cells to progenitor or even stem cell states [92-95]. Among immune cells, the B cell lineage itself has considerable plasticity [96,97]. B cells can be dedifferentiated by genetic manipulation to precursor cells, and thereafter diverted through intermediate states to fully differentiated T cells or macrophages [98,99]. Transiently expressing ectopic Bcl-6 and associated co-repressor MTA3 in plasma cell lines reversed the phenotype to that of B cells [100]. This finding is consistent with our model of the underlying bistable gene circuit, where TCDD first represses AP-1 and Blimp-1, leading to upregulation of Bcl-6 and Pax5, and consequent destabilization of the plasma cell state. While this prediction requires further study, it could have important implications for the immunotoxicity of TCDD. If confirmed, it would imply that besides suppressing initiation of the humoral immune response by inhibiting plasma cell formation, TCDD and similar compounds may disrupt the maintenance of the humoral response through destabilization of long-lived plasma cells.

Although the stochastic model presented here reproduces some experimental observations, the entire transcriptional network of B cell differentiation is potentially far more complex. Besides Bcl-6, Blimp-1, and Pax5, additional transcription factors such as Bach2, IRF-4, Mad1, and MTA3 are also involved in regulation of B-to-plasma cell differentiation [26,33,101-103]. Additional feedback loops may also exist, for example between Blimp-1 and IRF-4 [103]. The molecular pathways mediating the toxicity of TCDD are also likely to be more extensive than the impairment of AP-1 signaling. For instance, the role of the AhR repressor protein in TCDD signaling may need to be considered [104]. An important purpose of risk assessment for TCDD and other dioxin-like compounds is to establish specific "safe" values of exposure in the low-dose region, below which adverse biological effects are negligibly small. However, low-dose effects are generally more difficult to measure precisely and economically, as the variability associated with measured endpoints is usually large, obscuring the change in curvature of the low-dose region. Estimation of low-dose effects is therefore frequently made by extrapolation from high-dose data or computer simulations. In the present study, we show that a mechanistic, systems-level computational model of the B cell transcriptional network incorporating stochastic gene expression could generate dose response curves for a broad range of doses in agreement with experimental measurements. However, its value in accurately predicting low-dose effects for both LPS and TCDD is currently limited. A more confident prediction would likely require greater structural and kinetic details of pertinent signaling pathways leading from exposure to LPS and TCDD up to the bistable circuit, and perhaps a more complete description of the bistable circuit itself. Finally, for *in vivo *toxicity prediction, the cellular model has to be linked to dosimetry models that calculate the concentrations of dioxin-like compounds in immune tissues for environmental exposures.

In conclusion, we demonstrate that a bistable gene regulatory network comprising three genes - Bcl-6, Blimp-1, and Pax5 - exhibits behavior consistent with the terminal differentiation of B lymphocytes to antibody-secreting plasma cells. In a stochastic gene expression environment, the response to both antigen and TCDD at the cell population level appears to be substantially graded even though activation of individual cell is a binary, all-or-none phenomenon.

## Competing interests

The authors have declared that there are no conflicts of interest. This manuscript has been reviewed by the U. S. Environmental Protection Agency and approved for publication. Approval does not signify that the contents necessarily reflect the views and policies of the agency; nor does the mention of trade names or commercial products constitute endorsement or recommendation for use.

## Authors' contributions

All authors participated in the design of the *in silico *and *in vitro *experiments, and approved the final manuscript. QZ and SB developed the *in silico *model and performed numerical simulations. DEK and RBC^2 ^performed the *in vitro *experiments. QZ wrote the manuscript. SB, RBC^3^, RST, NEK, and MEA critically reviewed the manuscript.

## Supplementary Material

Additional file 1 **Supplementary Materials**. This file contains the ordinary differential equations, parameter values, and initial steady-state conditions for the B cell model presented in the main text, as well as additional figures from model simulations. Click here for file

Additional file 2 **B cell model in SBML format**. This file is the SMBL version of the B cell model presented in the main text. Click here for file
