# Supplementary material for: Stochastic Modeling of B Lymphocyte Terminal Differentiation and Its Suppression by Dioxin
Source: BMC Syst Biol. 2010 Apr 1;4:40. doi: 10.1186/1752-0509-4-40 (PMC2859749; doi:10.1186/1752-0509-4-40)
Supplement: Additional file 1 — Supplementary Materials. This file contains the ordinary differential equations, parameter values, and initial steady-state conditions for the B cell model presented in the main text, as well as additional figures from model simulations. [file 1752-0509-4-40-S1.PDF]

Supplementary Materials

Stochastic Modeling of B Lymphocyte Terminal Differentiation and Its Suppression by Dioxin

Qiang Zhang, Sudin Bhattacharya, Douglas E. Kline, Robert B. Crawford, Rory B. Conolly, Russell S. Thomas, Norbert E. Kaminski, and Melvin E. Andersen

Table S1. Ordinary differential equations for the molecular species illustrated in Figure S1

$$\frac{d(Bcl6\_GENE0)}{dt} = (k_{00} + \frac{k_{01} \cdot Blimp1}{Kd_{01} + Blimp1}) \cdot Bcl6\_GENE1 - k_{02} \cdot Bcl6\_GENE0$$

$$\frac{d(Bcl6\_GENE1)}{dt} = -(k_{00} + \frac{k_{01} \cdot Blimp1}{Kd_{01} + Blimp1}) \cdot Bcl6\_GENE1 + k_{02} \cdot Bcl6\_GENE0$$

$$\frac{d(Bcl6\_mRNA)}{dt} = k_{03} \cdot Bcl6\_GENE1 - k_{04} \cdot Bcl6\_mRNA$$

$$\frac{d(Bcl6)}{dt} = k_{05} \cdot Bcl6\_mRNA - k_{06} \cdot Bcl6$$

$$\frac{d(Blimp1\_GENE0)}{dt} = \frac{k_{11} \cdot Pax5}{kd_{11} + Pax5} \cdot Blimp1\_GENE1 - (k_{10} + k_{12} \cdot \frac{AP1p}{Kd_{13} + AP1p} \cdot \frac{Kd_{12}}{Kd_{12} + Bcl6}) \cdot Blimp1\_GENE0$$

$$\frac{d(Blimp1\_GENE1)}{dt} = -\frac{k_{11} \cdot Pax5}{kd_{11} + Pax5} \cdot Blimp1\_GENE1 + (k_{10} + k_{12} \cdot \frac{AP1p}{Kd_{13} + AP1p} \cdot \frac{Kd_{12}}{Kd_{12} + Bcl6}) \cdot Blimp1\_GENE0$$

$$\frac{d(Blimp1\_mRNA)}{dt} = k_{13} \cdot Blimp1\_GENE1 - k_{14} \cdot Blimp1\_mRNA$$

$$\frac{d(Blimp1)}{dt} = k_{15} \cdot Blimp1\_mRNA - k_{16} \cdot Blimp1$$

$$\frac{d(Pax5\_GENE0)}{dt} = (k_{20} + \frac{k_{21} \cdot Blimp1}{Kd_{21} + Blimp1}) \cdot Pax5\_GENE1 - k_{22} \cdot Pax5\_GENE0$$

$$\frac{d(Pax5\_GENE1)}{dt} = -(k_{20} + \frac{k_{21} \cdot Blimp1}{Kd_{21} + Blimp1}) \cdot Pax5\_GENE1 + k_{22} \cdot Pax5\_GENE0$$

$$\frac{d(Pax5\_mRNA)}{dt} = k_{23} \cdot Pax5\_GENE1 - k_{24} \cdot Pax5\_mRNA$$

$$\frac{d(Pax5)}{dt} = k_{25} \cdot Pax5\_mRNA - k_{26} \cdot Pax5$$

$$\frac{d(AP1)}{dt} = k_{31} \cdot \frac{Kd_{31}}{Kd_{31} + TCDD\_AhR} - k_{32} \cdot AP1 + k_{33} \cdot AP1p - (k_{34} + k_{35} \cdot LPS\_TLR4) \cdot AP1$$

$$\frac{d(AP1p)}{dt} = -k_{33} \cdot AP1p + (k_{34} + k_{35} \cdot LPS\_TLR4) \cdot AP1 - k_{32} \cdot AP1$$

$$\frac{d(AhR)}{dt} = -k_{41} \cdot TCDD \cdot AhR + k_{42} \cdot TCDD\_AhR$$

$$\frac{d(TCDD\_AhR)}{dt} = k_{41} \cdot TCDD \cdot AhR - k_{42} \cdot TCDD\_AhR$$

$$\frac{d(TLR4)}{dt} = k_{51} - k_{52} \cdot TLR4 - k_{53} \cdot LPS \cdot TLR4 + k_{54} \cdot LPS\_TLR4$$

$\frac{d(LPS\_TLR4)}{dt}$	=	$k_{53} \cdot LPS \cdot TLR4 - k_{54} \cdot LPS\_TLR4 - \frac{k_{55} \cdot LPS\_TLR4}{Kd_{51} + LPS\_TLR4}$
$\frac{d(IgM)}{dt}$	=	$k_{61} \cdot \frac{Kd_{61}^{2}}{Kd_{61}^{2} + Pax5^{2}} \cdot \frac{Kd_{62}}{Kd_{62} + TCDD\_AhR} - k_{62} \cdot IgM$
$\frac{d(IgMs)}{dt}$	=	$k_{62} \cdot IgM$

Note: Refer to Figure S1 legend for variable names used for different molecular species.

Table S2. Parameter values for the model illustrated in Figure S1

Parameter	Value	Unit	Note
k_{00}	0.042	s^{-1}	These parameters are associated with the gene activation and deactivation of Bcl6, Blimp1, and Pax5. They are adjusted to achieve desired bistability as indicated in Figure 2A in the main text. We assume that the kinetics of gene activation and deactivation are faster than that of mRNA and protein degradation so that the time required for completion of the bistable switching once initiated depends more on the latter.<br>Specifically, k_{20} and k_{21}, in association with k_{22}, set the high and low ends (responses) of the Pax5 null curve, respectively; Kd_{21} sets the ED50 of the curve. Similarly, k_{00}, k_{01}, k_{02}, and Kd_{01} specify the Bcl6 null curve. With respect to the Blimp1 null curve, k_{10}, k_{11}, and k_{12} set the low and high ends of the curve; Kd_{11} sets the ED50, and Kd_{12} plays a role in the steepness of the Blimp1 null curve.<br>Taken together, the values of these parameters were adjusted to give rise to the null curves in Figure 2A, which represent a robust bistable switch.
k_{10}	6.5e-5	s^{-1}	
k_{20}	0.042	s^{-1}	
k_{01}	39.3	s^{-1}	
k_{11}	0.08	s^{-1}	
k_{21}	39.3	s^{-1}	
k_{02}	0.01	s^{-1}	
k_{12}	2.01	s^{-1}	
k_{22}	0.01	s^{-1}	
Kd_{01}	1e3	-	
Kd_{11}	150	-	
Kd_{12}	1.11	-	
Kd_{21}	1e3	-	
k_{03}	0.0425	s^{-1}	These transcription rate constants (k_{03}, k_{13}, and k_{23}) and translation rate constants (k_{05}, k_{15}, and k_{25}) together determine the upper limits of the protein levels of Bcl6, Blimp1, and Pax5. Their ratios are adjusted so that noise in protein expression is not too high to cause significant spontaneous switching between the B cell and plasma cell states. k_{13} and k_{15}, responsible for Blimp1 transcription and translation, are also varied to investigate the effect of Blimp1 noise on the steepness of dose response curves (see Figure 7 in the main text).
k_{13}	0.012	s^{-1}	
k_{23}	0.0425	s^{-1}	
k_{05}	6.0e-4	s^{-1}	
k_{15}	6.0e-4	s^{-1}	
k_{25}	6.0e-4	s^{-1}	
k_{04}	1.93e-4	s^{-1}	Under antigen stimulation, antibody-secreting plasma cells can appear as early as 24 h [1-4]. This suggests that the half-lives of Bcl6, Blimp1, and Pax5 mRNAs and proteins, which comprise the bistable switch, have to be considerably less than 24 h – given that the half-lives determine to a large extent the time it takes to reach a new steady state. Thus we assumed that k_{04}, k_{14}, and k_{24} all have a value of 1.93e-4 s^{-1}, corresponding to a half-life of 1 h for the Bcl6, Blimp1, and Pax5 mRNAs. Together with the parameters defining the half-lives for the proteins, the relatively short half-lives allow the bistable switching per se to be a fast process.
k_{14}	1.93e-4	s^{-1}	
k_{24}	1.93e-4	s^{-1}	
k_{06}	9.65e-5	s^{-1}	These values yield 2-h half-lives for Bcl6 and Pax5, and 1-h half-life for Blimp1. Together with the parameters defining the half-lives for the mRNAs, the relatively short half-lives allow the bistable switching per se to be a fast process.
k_{16}	1.93e-4	s^{-1}	
k_{26}	9.65e-5	s^{-1}	
Kd_{13}	1e4	-	This parameter affects the degree of activation of Blimp1 gene by AP1p. Its vale is adjusted so that the maximal percentage plasma cells formation at 72 h is about 10% of the B cell population according to the experimental data (see Figure 6 in the main text).
k_{31}	4.02e-3	s^{-1}	These two parameter values give rise to a total AP1 level of

k_{32}	8.04e-6	s^{-1}	500, and a half-life of 24 h is assumed for AP1.
k_{33}	1.25e-3	s^{-1}	These two parameters set the basal dephosphorylation and phosphorylation rates for AP1 and AP1p. At the chosen values, the basal AP1p is 100 and AP1 is 400. The latter is the pool of AP1 that can be readily phosphorylated in response to LPS stimulation.
k_{34}	3.146e-4	s^{-1}	
k_{35}	5.9e-6	s^{-1}	This parameter is responsible for LPS-stimulated AP1 phosphorylation. Its value is chosen so that the maximal LPS-stimulated increase in AP1p is about 4-fold of the basal level, which is consistent with the >3-fold activation observed experimentally [5, 6].
Kd_{31}	1.125e4	-	This value is chosen so that TCDD can maximally inhibit plasma cell formation and IgM secretion to about 1/3 of the control as reported earlier [7-9] and also demonstrated here (see Figure S4).
k_{41}	4e-4	$nM^{-1}s^{-1}$	This pair of values are chosen to yield a dissociation constant of 5 nM for TCDD and AhR binding, in line with the reported range of 0.8 – 14 nM [10-12]. The dissociation rate constant k_{42} also makes the binding kinetics occur on a relatively fast time scale, compatible to ligand such as 17β-estradiol binding to the estrogen receptor [13, 14].
k_{42}	2e-3	s^{-1}	
k_{51}	1.93e-3	s^{-1}	This pair of values are chosen to yield a basal TLR4 level of 1000. A relative long half-life (100 h, determined by k_{52}) for free TLR4 is used, which along with k_{55} and Kd_{51}, allows the downregulation of LPS-bound TLR4 to occur in a time frame of a couple of days.
k_{52}	1.93e-6	s^{-1}	
k_{53}	2.08e-4	$ml \cdot \mu g^{-1}s^{-1}$	For an average molecular weight of 8 kDa for LPS, values chosen for k_{53} and k_{54} together yield a dissociation constant of 63 nM for LPS binding to TLR4, which is close to the reported value of 65 nM [15].
k_{54}	1.0e-4	s^{-1}	
k_{55}	0.012	s^{-1}	These two parameters are responsible for TLR4 downregulation upon LPS binding. Their values are chosen so that AP1p activation is attenuated over time and returns to the basal level by 72 h as observed experimentally [5, 6]. This attenuation profile, as shown in Figure 4 in the main text, is in turn responsible for the temporal changes in plasma cell formation and IgM secretion shown in Figure 6.
Kd_{51}	50	-	
k_{61}	0.107	s^{-1}	Values of these two parameters determine that the maximal IgM in a plasma cell is about 1000. k_{62} sets the rate constant at which intracellular IgM is secreted. With this value, it is assumed that it takes 2 h for half of the intracellular IgM content to be secreted.
k_{62}	9.625e-5	s^{-1}	
Kd_{61}	9	-	This parameter value determines that when Pax5 is highly expressed as in the B cell state, IgM is suppressed to a basal level of 1.
Kd_{62}	3e4	-	This parameter accounts for a small amount of direct inhibition of IgM production by TCDD [16].
LPS	0	μg/ml	The concentration is varied for dose response curves.
TCDD	0	nM	The concentration is varied for dose response curves.

Note: Most parameter values, especially those specific to the B cell, are unknown. They are either

assumed based on similar processes or general knowledge from the literature, or estimated by constraining the model with relevant experimental data from the literature or of our own. For simplicity, parameters associated with Bcl6 and Pax5 gene activation/deactivation, transcription, translation, and degradation are made identical for these two genes.

Table S3. Initial steady-state levels of all molecular species shown in Figure S1

Molecular Species	Initial Steady-State Value	
	**B cell state**	**Plasma cell state**
Bcl6_GENE0	1.78	1.9978
Bcl6_GENE1	0.22	0.0022
Bcl6_mRNA	48	0.48
Bcl6	300	3
Blimp1_GENE0	1.99	0.45
Blimp1_GENE1	0.01	1.55
Blimp1_mRNA	0.32	96.5
Blimp1	1	300
Pax5_GENE0	1.78	1.9978
Pax5_GENE1	0.22	0.0022
Pax5_mRNA	48	0.48
Pax5	300	3
AP1	400	400
AP1p	100	100
AhR	10000	10000
TCDD_AhR	0	0
TLR4	1000	1000
LPS_TLR4	0	0
IgM	1	1000
IgMs	0	0

Note:

1. Each value represents number of molecules per cell. For the stochastic simulations, these values were rounded to the nearest integers which were then used as the initial condition. For both deterministic and stochastic simulations, the model was first allowed to stabilize for at least 200 hours from assigned initial conditions before any LPS and/or TCDD treatment were applied.

2. Steady-state values for most of the molecular species in the model are unknown and were thus assumed. Two copies were ascribed to each gene. The reported intracellular AhR abundance ranges from 5,000 to 323,000 molecules for a variety of cell types [10-12, 17]; we used a value of 10,000 in the model.

3. The values of Bcl6, Blimp1, and Pax5 protein levels for the B cell state and plasma cell state were established based on the following assumptions. For many transcription factors involved in cell differentiation and exhibiting a binary gene expression pattern, it is reasonable to assume that the high-expressing (On) and low-expressing (Off) levels are separated by two-order of magnitude or greater. With respect to Blimp1, it has been shown that its protein expression level in plasma cells is several hundred-fold higher than that in B cells [18]. We further assume that when in the Off state, a transcription factor in the cell has only a few copy numbers or less. Then when in the On state, the abundance will be in the range of a few hundreds of copy numbers. With such a configuration, the B cell and plasma cell states, marked by the differential expression levels of the three transcription factors, are clearly separated, and spontaneous switching between the two states due to stochastic fluctuations occurs only rarely.

4. Protein molecules involved in a signal transduction pathway such as TLR4 and AP1 could exist in relatively high abundance. Stochastic simulations are computationally intensive and the more molecules present in the system, the more computational time required. Relatively low values for TLR4, AP1, and AP1p were used to ensure no significant slow-down in simulation. In order for this downscaling not to affect the model behavior tangibly, the parameters associated with the downstream actions of LPS-bound TLR4 (k_{35}) and AP1p (Kd_{13}) are also rescaled accordingly to “offset” the effects

of downscaled abundance of the variables. Moreover, still at levels above a hundred copy numbers, there are no significant stochastic fluctuations in these downscaled variables, as exemplified by the AP-1p levels in Figure 4 in the main text. In the model IgM is largely a readout of the state of the bistable circuit. So its absolute value should not affect the behavior of the circuit. Although the actually level of IgM in a plasma cell could be much higher than used here, we limited the maximum to 1000 to avoid significant slow-down in simulation.

5. A simulated cell is considered as a plasma cell when its IgM level exceeds 200. The variable IgMs, which corresponds to the amount of IgM secreted into the cell culture medium, was set to 0 at the onset of each LPS and/or TCDD treatment.

6. Refer to Figure S1 legend for variable names used for different molecular species.

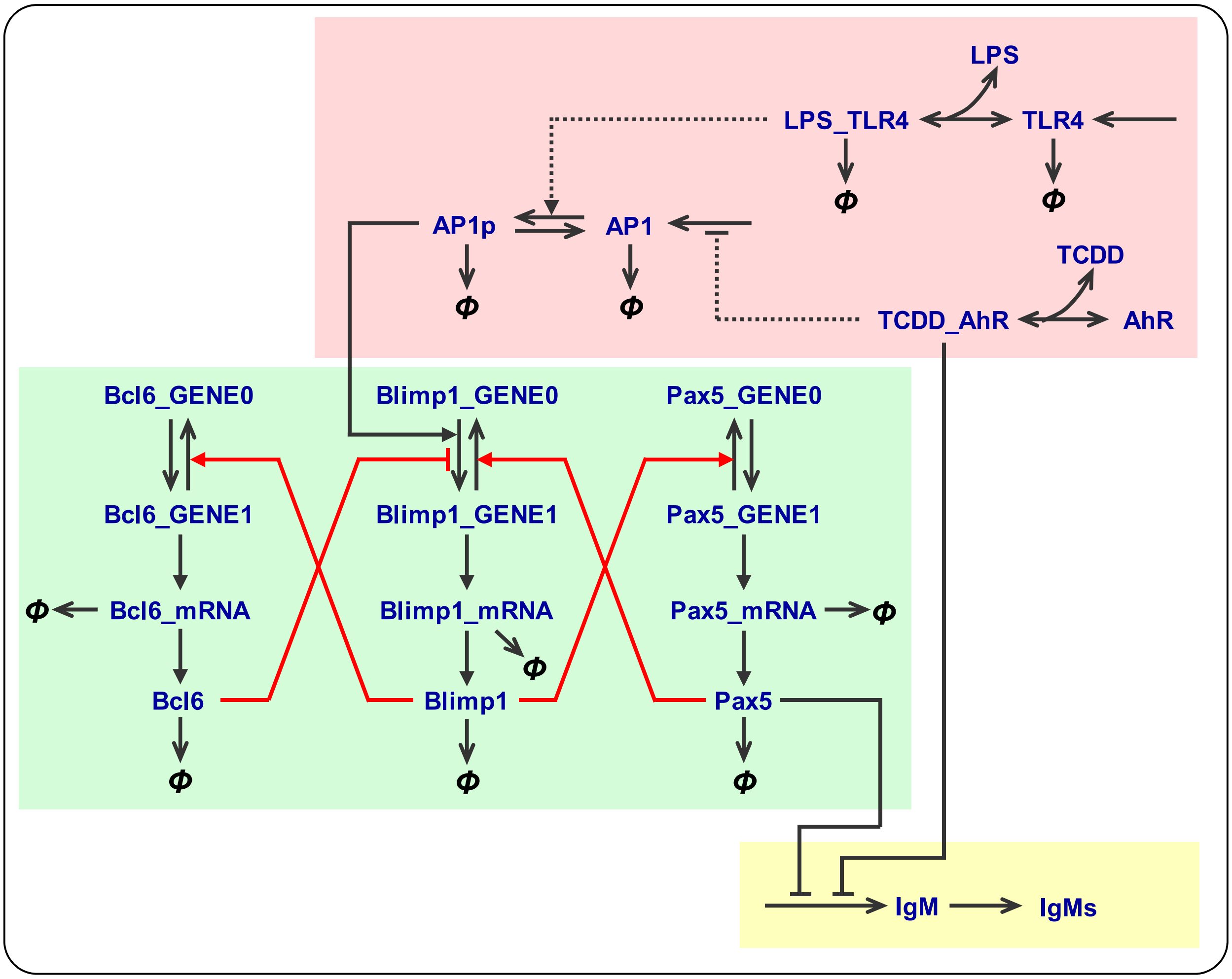

Figure S1. Structure of the transcriptional network model underlying LPS-stimulated B cell terminal differentiation and its suppression by TCDD. The ordinary differential equations describing the interactions, parameter values, and initial steady-state values are listed in Tables S1, S2 and S3, respectively. The entities Bcl6_GENE0, Blimp1_GENE0, and Pax5_GENE0 denote the inactive states of Bcl-6, Blimp-1, and Pax5 genes; Bcl6_GENE1, Blimp1_GENE1, and Pax5_GENE1 represent the active states of the corresponding genes. LPS_TLR4: LPS and TLR4 receptor complex; TCDD_AhR: TCDD and AhR complex; AP1p: transcriptionally active AP-1. IgM: intracellular immunoglobulin M; IgMs: secreted IgM. Φ denotes mRNA or protein degradation. Arrows with dashed line represent indirect interactions.

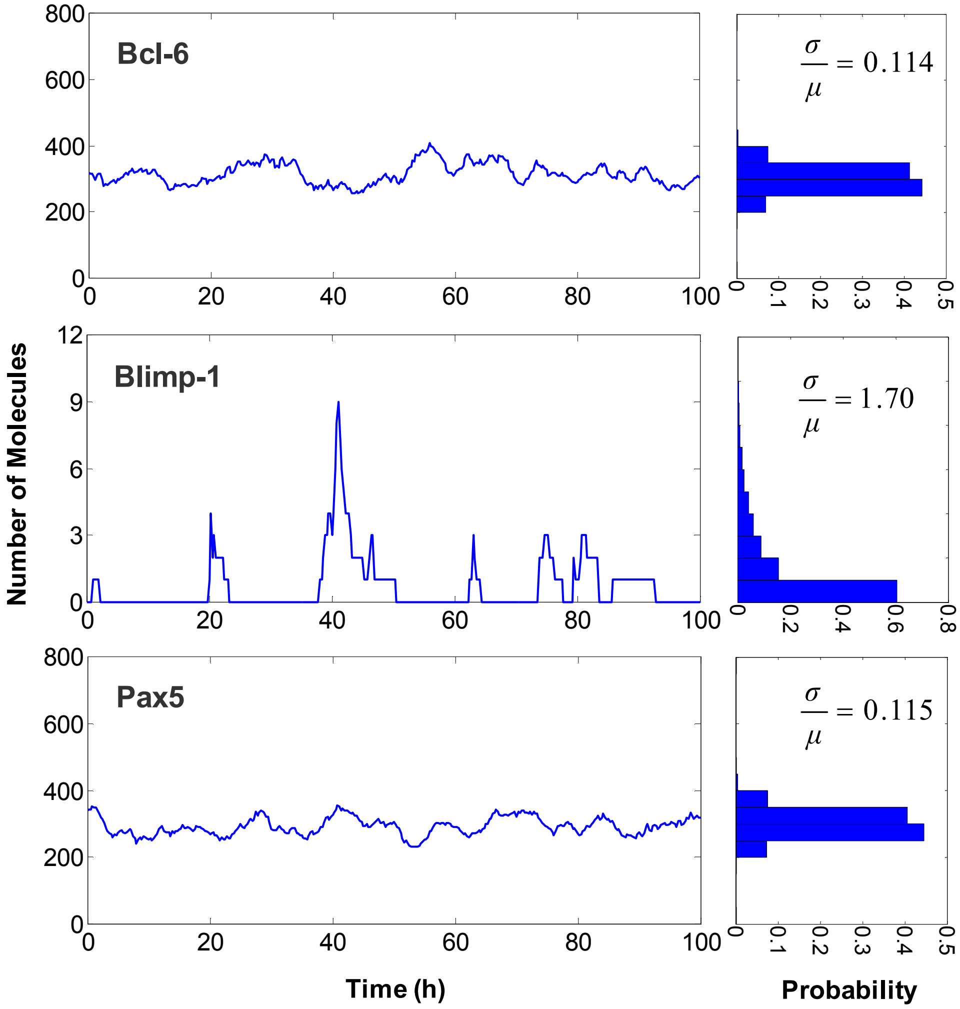

Figure S2. Stochastic gene expression of Bcl-6, Blimp-1, and Pax5 with the repressive input to Bcl-6 and Pax5 from Blimp-1 replaced by its deterministic mean in the B cell state. The noise level is quantified by the coefficient of variation, σ/μ (where σ is the standard deviation and μ the mean of the protein level of each gene). The histograms were obtained from 10^5 simulated cells. Compared with Figure 3 in the main text, the noise in Bcl-6 and Pax5 gene expression is dramatically reduced, while the noise in Blimp-1 gene expression is only reduced slightly. This indicates that most of the fluctuations in Bcl-6 and Pax5 gene expression in the full model originate from stochastic Blimp-1 gene expression.

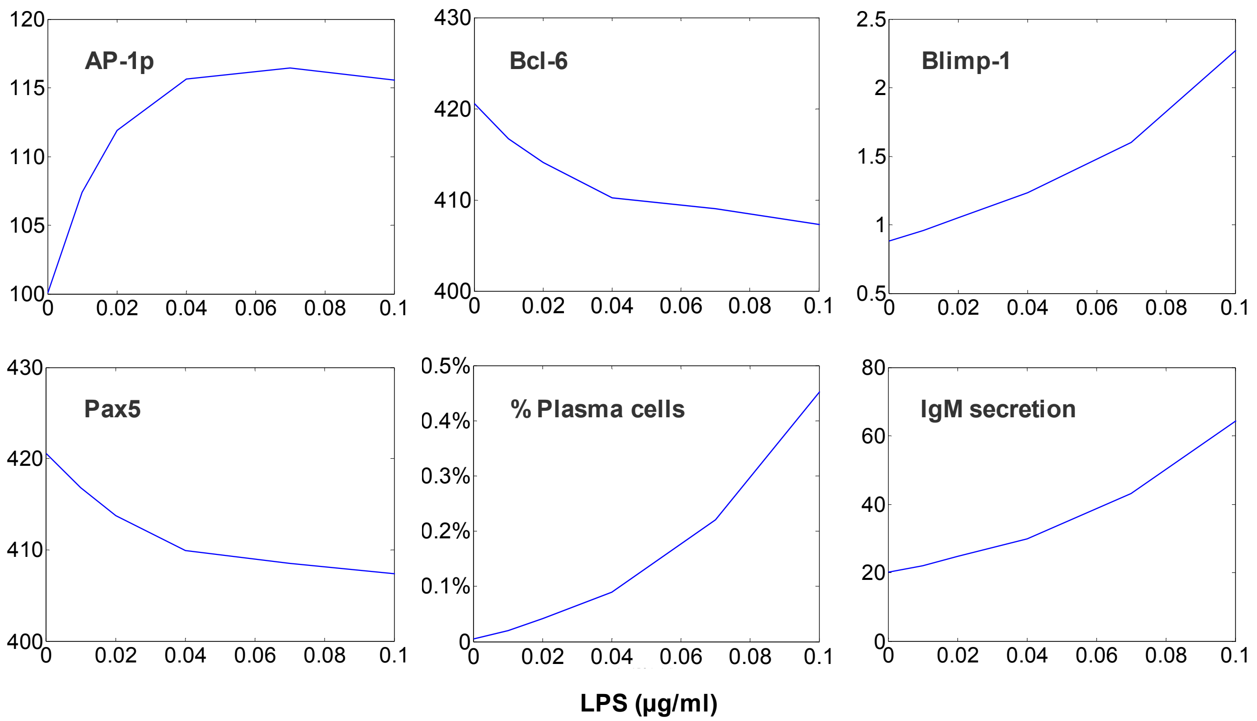

Figure S3. Simulated low-dose effect of LPS on B cell differentiation. The results were obtained from 10^5 simulated cells under continuous LPS stimulation for 72 h. Except for percentage of plasma cell formation (middle panel on bottom), all other responses (AP-1p, Bcl-6, Blimp-1, Pax5, and secreted IgM) are presented as averaged copy numbers per cell.

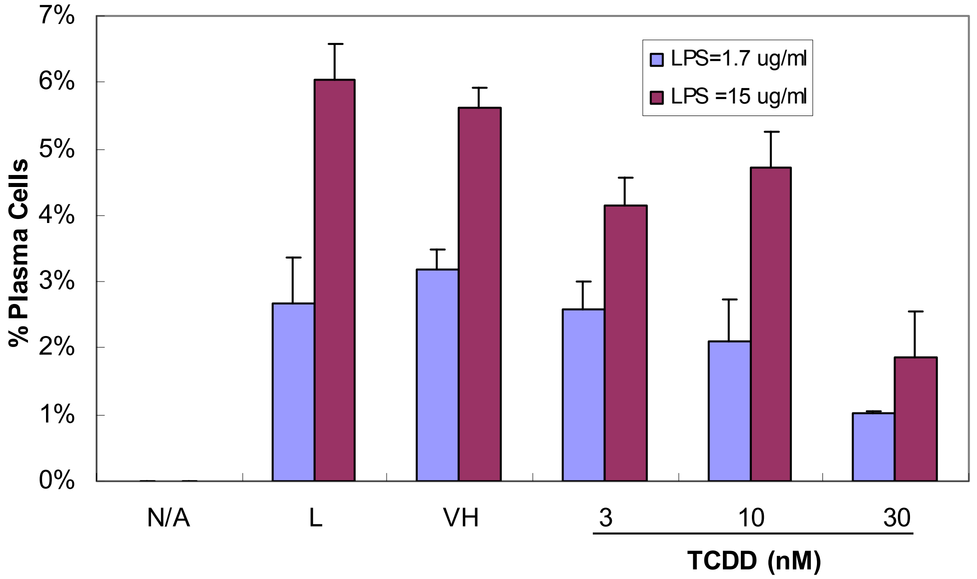

Figure S4. Experimental results on suppression of LPS-stimulated plasma cell formation by TCDD. N/A: naive B cells; L: LPS alone; VH: LPS + vehicle (0.01% DMSO). Vehicle or TCDD was added to primary B cell cultures at the same time as LPS was added. Cell cultures were than incubated for 72 h. Each bar represents triplicate samples of primary B cells.

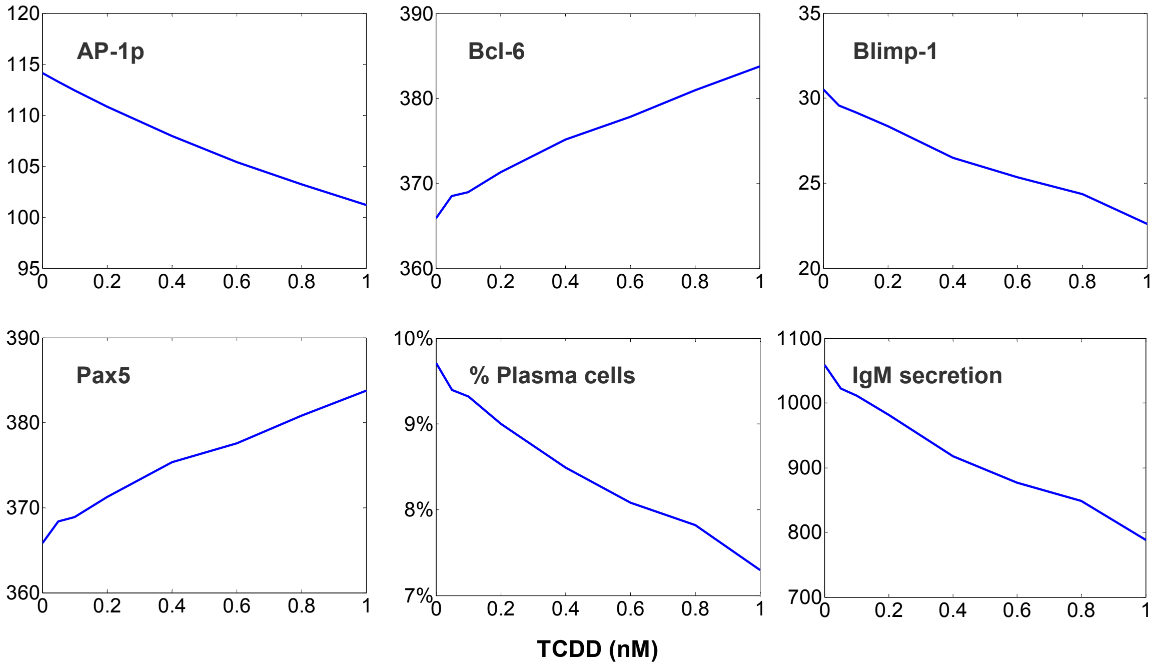

Figure S5. Simulated suppressive effect of TCDD at low doses on LPS-stimulated B cell differentiation. The results were obtained from 10^5 simulated cells under continuous LPS (15μg/ml) and TCDD treatment for 72 h. Except for the percentage plasma cell response (middle panel on bottom), other responses (AP-1p, Bcl-6, Blimp-1, Pax5, and secreted IgM) are averaged copy numbers per cell.

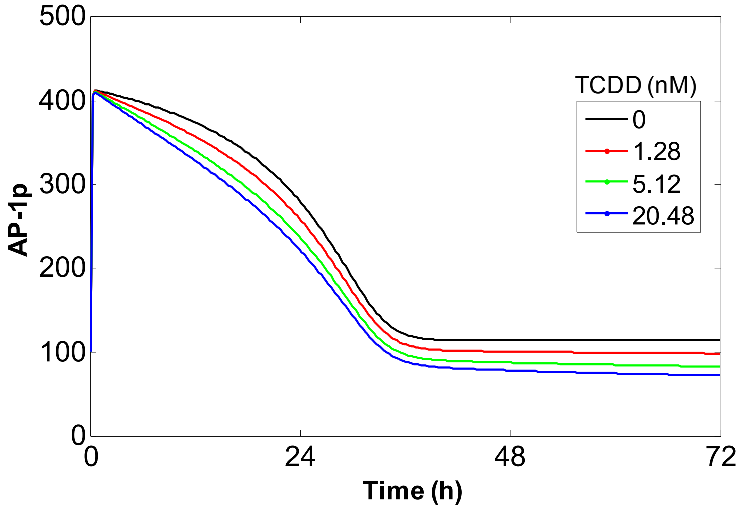

Figure S6. Deterministic simulation of suppressive effect of TCDD on AP-1 activation. LPS concentration used is 15 μg/ml.

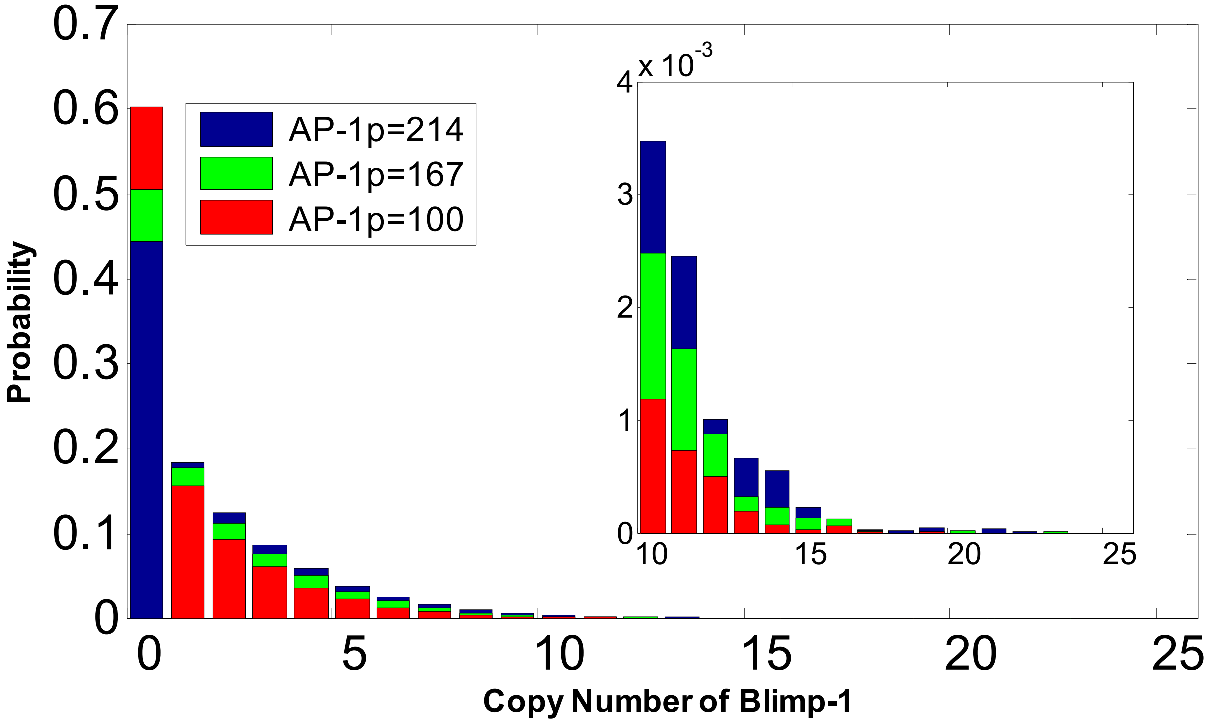

Figure S7. Histograms of Blimp-1 protein level induced by AP-1p fixed at various levels. To remove the compounding effects of the feedback loops on Blimp-1 protein noise, the repressive inputs from Bcl-6 and Pax5 were replaced by their deterministic means (300 for both). The histograms were obtained from 10^5 simulated cells. AP-1p levels indicated were obtained by setting k34 (basal AP-1 phosphorylation rate constant) to 1, 2, and 3-fold of the default value, respectively. The resulting mean and noise level (coefficient of variation) of Blimp-1 protein are 1.00, 1.70; 1.35, 1.47; and 1.60, 1.35, respectively. As the mean of Blimp-1 protein increases due to increase in AP-1p, pulses of higher magnitude, such as those with copy number greater than 10 at peak, are more frequently observed (inset). More frequent and larger Blimp-1 pulses are responsible for increased probability of bistable switching at higher LPS concentrations.

References

1. Soro PG, Morales-A P, Martinez-M JA, Morales-A S, Copin SG, Marcos MAR, Gaspar ML: Differential involvement of the transcription factor Blimp-1 in T cell-independent and -dependent B cell differentiation to plasma cells. *Journal of Immunology* 1999, 163(2):611-617.
2. Rui L, Healy JI, Blasioli J, Goodnow CC: ERK signaling is a molecular switch integrating opposing inputs from B cell receptor and T cell cytokines to control TLR4-driven plasma cell differentiation. *J Immunol* 2006, 177(8):5337-5346.
3. Ohkubo Y, Arima M, Arguni E, Okada S, Yamashita K, Asari S, Obata S, Sakamoto A, Hatano M, O-Wang J *et al*: A role for c-fos/activator protein 1 in B lymphocyte terminal differentiation. *Journal of Immunology* 2005, 174(12):7703-7710.
4. Swain SL, Dutton RW, McKenzie D, Helstrom H, English M: Role of antigen in the B cell response. Specific antigen and the lymphokine IL-5 synergize to drive B cell lymphoma proliferation and differentiation to Ig secretion. *J Immunol* 1988, 140(12):4224-4230.
5. Ohkubo Y, Arima M, Arguni E, Okada S, Yamashita K, Asari S, Obata S, Sakamoto A, Hatano M, J OW *et al*: A role for c-fos/activator protein 1 in B lymphocyte terminal differentiation. *J Immunol* 2005, 174(12):7703-7710.
6. Suh J, Jeon YJ, Kim HM, Kang JS, Kaminski NE, Yang KH: Aryl hydrocarbon receptor-dependent inhibition of AP-1 activity by 2,3,7,8-tetrachlorodibenzo-p-dioxin in activated B cells. *Toxicol Appl Pharmacol* 2002, 181(2):116-123.
7. Tucker AN, Vore SJ, Luster MI: Suppression of B cell differentiation by 2,3,7,8-tetrachlorodibenzo-p-dioxin. *Mol Pharmacol* 1986, 29(4):372-377.
8. North CM, Kim BS, Snyder N, Crawford RB, Holsapple MP, Kaminski NE: TCDD-Mediated Suppression of the in vitro Anti-Sheep Erythrocyte IgM Antibody Forming Cell Response is Reversed by Interferon Gamma. *Toxicol Sci* 2008.
9. Schneider D, Manzan MA, Crawford RB, Chen W, Kaminski NE: 2,3,7,8-Tetrachlorodibenzo-p-dioxin-mediated impairment of B cell differentiation involves dysregulation of paired box 5 (Pax5) isoform, Pax5a. *J Pharmacol Exp Ther* 2008, 326(2):463-474.
10. Roberts EA, Harper PA, Wong JM, Wang Y, Yang S: Failure of Ah receptor to mediate induction of cytochromes P450 in the CYP1 family in the human hepatoma line SK-Hep-1. *Arch Biochem Biophys* 2000, 384(1):190-198.
11. Roberts EA, Johnson KC, Dippold WG: Ah receptor mediating induction of cytochrome P450IA1 in a novel continuous human liver cell line (Mz-Hep-1). Detection by binding with [3H]2,3,7,8-tetrachlorodibenzo-p-dioxin and relationship to the activity of aryl hydrocarbon hydroxylase. *Biochem Pharmacol* 1991, 42(3):521-528.
12. Roberts EA, Johnson KC, Harper PA, Okey AB: Characterization of the Ah receptor mediating aryl hydrocarbon hydroxylase induction in the human liver cell line Hep G2. *Arch Biochem Biophys* 1990, 276(2):442-450.
13. Rich RL, Hoth LR, Geoghegan KF, Brown TA, LeMotte PK, Simons SP, Hensley P, Myszka DG: Kinetic analysis of estrogen receptor/ligand interactions. *Proc Natl Acad Sci U S A* 2002, 99(13):8562-8567.
14. Weichman BM, Notides AC: Estradiol-binding kinetics of the activated and nonactivated estrogen receptor. *J Biol Chem* 1977, 252(24):8856-8862.
15. Viriyakosol S, Tobias PS, Kitchens RL, Kirkland TN: MD-2 binds to bacterial lipopolysaccharide. *J Biol Chem* 2001, 276(41):38044-38051.
16. Sulentic CE, Zhang W, Na YJ, Kaminski NE: 2,3,7,8-tetrachlorodibenzo-p-dioxin, an exogenous modulator of the 3'alpha immunoglobulin heavy chain enhancer in the CH12.LX mouse cell line. *J Pharmacol Exp Ther* 2004, 309(1):71-78.
17. Holmes JL, Pollenz RS: Determination of aryl hydrocarbon receptor nuclear translocator protein concentration and subcellular localization in hepatic and nonhepatic cell culture lines: development of quantitative Western blotting protocols for calculation of aryl hydrocarbon receptor and aryl hydrocarbon receptor nuclear translocator protein in total cell lysates. *Mol Pharmacol* 1997, 52(2):202-211.
18. Fairfax KA, Corcoran LM, Pridans C, Huntington ND, Kallies A, Nutt SL, Tarlinton DM: Different kinetics of blimp-1 induction in B cell subsets revealed by reporter gene. *J Immunol* 2007, 178(7):4104-4111.
